# Survivability of Suddenly Loaded Arrays of Micropillars

**DOI:** 10.3390/ma14237173

**Published:** 2021-11-25

**Authors:** Tomasz Derda, Zbigniew Domanski

**Affiliations:** Department of Mathematics, Czestochowa University of Technology, PL-42-201 Czestochowa, Poland; zbigniew.domanski@pcz.pl

**Keywords:** arrays of micropillars, failure avalanches, fibre bundle model, piezoeletric nanogenerators, statistics, survivability

## Abstract

When a multicomponent system is suddenly loaded, its capability of bearing the load depends not only on the strength of components but also on how a load released by a failed component is distributed among the remaining intact ones. Specifically, we consider an array of pillars which are located on a flat substrate and subjected to an impulsive and compressive load. Immediately after the loading, the pillars whose strengths are below the load magnitude crash. Then, loads released by these crashed pillars are transferred to and assimilated by the intact ones according to a load-sharing rule which reflects the mechanical properties of the pillars and the substrate. A sequence of bursts involving crashes and load transfers either destroys all the pillars or drives the array to a stable configuration when a smaller number of pillars sustain the applied load. By employing a fibre bundle model framework, we numerically study how the array integrity depends on sudden loading amplitudes, randomly distributed pillar strength thresholds and varying ranges of load transfer. Based on the simulation, we estimate the survivability of arrays of pillars defined as the probability of sustaining the applied load despite numerous damaged pillars. It is found that the resulting survival functions are accurately fitted by the family of complementary cumulative skew-normal distributions.

## 1. Introduction

Significant progress has been made in the creation and development of sub-micron-scale devices, including nanopillars assembled in an ordered fashion on flat substrates [1,2]. Nowadays, mezo-scale pillar arrays are applied in areas of nanotechnology such as bio-mechanical sensing [3], nanoscale electronics, photovoltaics or thermoelectrics, to name a few [4,5,6]. An interesting class of nanodevices includes high-performance piezoelectric nanogenerators (NG) that convert external mechanical energy into electricity [7]. The newest family of these energy-harvesting devices consists of NGs with vertical core–shell micropillars [7,8] stacked between flexible flat substrates that play the role of base and counter electrodes. When an external load is applied axially in a form of loading-hold-unloading pulses, the counter electrode moves correspondingly, and the alternating current is generated due to the piezoelectric effect. Flexible sensors that are capable of measuring multidirectional forces represent another group of devices involving arrays of micropillars. In this case, micropillars that are aligned vertically and sandwiched between electrodes form a piezoelectric sensing unit capable of detecting a normal compressive force [9].

Small-scale structures have a substantially greater surface area to volume ratio compared to their coarse-grained counterparts [10]. When a nanopillar is slightly compressed, bulk defects (mostly dislocations) move and disappear at the surface. As a result, there are not enough active defects in the bulk to induce deformations [11]. Multiple uniaxial tensile and compressive experiments on metallic nanopillars confirm the substantial strength increase via the size reduction of the sample [12,13,14]. The knowledge of even the most accurate estimate of the strength of individual pillars, however, does not suffice in predicting the overall strength of these pillars when they work together after being assembled into an array. To illustrate this known fact, take a pillar whose overall capability of correctly bearing a load is characterized by the strength threshold σ. Then, consider a system composed of a multitude of such pillars which are functionally identical but differ in values of σ. When the system is loaded, pillars whose σ values are smaller than loads exerted locally on them fail. The local loads from these failing pillars are transferred to ones that remain intact. It turns out that the ultimate strength of the system crucially depends on how such a transfer happens [15,16,17], what the topology of interconnections among components is [18], what the loading conditions are [19,20], and whether the loading is applied step-wise, suddenly [21] or cyclically [22,23]. This means that a given ensemble of interconnected components, working together along with an established rule of load transfer between failed and intact components, may either sustain an externally applied load or become entirely destroyed if an unsuitable rule governs the load transfer or interconnections switch to unfavourable topology [18,24].

In this work, we restrict ourselves to arrays of pillars viewed as mechanical systems. From this perspective, the strength and integrity of a given array mainly depend on aspects such as mutual pillar arrangements, pillar-strength thresholds, a chosen method of loading and the load-transfer rule. Among them the method of loading and the load-transfer rule are of particular importance.

Although a variety of schemes of loading are discussed in the literature, the quasi-static and sudden loadings are especially well-studied. The quasi-static scheme is comprised of many small step-like load increases, whereas in the sudden loading, the applied load rises abruptly from zero to a given magnitude *F*. While these schemes are conceptually similar, the final states of a specified loaded array are quite different. At the very beginning of a quasi-static loading, only the weakest pillars fail. Their loads are distributed among intact pillars and the array smoothly passes to a stable configuration when a slightly smaller number of pillars support the applied load. Then, the subsequent load values are adjusted to break the weakest existing pillars and settle the system in the resulting stable configurations. Due to such a sequence of steps comprised of pillar-breaking and load redistributions, the system moves between consecutive accessible stable configurations until a presumed value *F* of the applied load is reached. A different scenario emerges when the same array of *N* pillars is suddenly loaded and the load amplitude f=F/N surmounts strength thresholds σ of weak pillars. The pillars whose σ≤f are crushed instantly after the load appears and a rather large amount of loads are transferred to pillars characterised by σ>f. Hence, a non-negligible fraction of these pillars become overloaded triggering consecutive cascades of pillar failures and resulting load transfers. After a certain number of such cascades, the array either collapses or freezes in a stable configuration which is different from the one reached under the quasi-static loading.

The principal objective of this work is to model and quantify the relationship between the load applied suddenly to the pillars and the integrity of the array of pillars itself. Specifically, we focus on the survivability of suddenly loaded arrays understood as the capability to sustain the applied load despite the partial damage. For this purpose, we simulated the loading process and, based on the resulting statistics, we estimated the corresponding survival function *S*. In our approach, *S* represents the probability that a given array of initially *N* vertical pillars bears the axial load F=Nf in the presence of a number n<N of intact pillars reduced because of crushing.

In Section 2, we specify our model together with corresponding computational scheme. Section 3 presents the results of simulations and a discussion. Finally, we summarize our findings and formulate conclusions related to the integrity of a suddenly loaded array of pillars.

## 2. Model Description and Computational Scheme

An array of our interest consists of *N* parallel pillars positioned vertically at N=L×L nodes of square flat substrate. An axial and suddenly applied load induces pillar crushes which spread through the array. To make a link between the applied load and evolving failures, we employ the fibre bundle model framework [25,26,27,28], which enables us to independently tune the main aspects of our rough model.

### 2.1. Pillars

While we neglect a possible pillar-alignment mismatch, the pillars themselves contain imperfections either due to fabrication errors or due to inherent material defects. In order to include these imperfections in the model, we represent the pillars by a quenched set of random-load thresholds $\left\{\sigma_{th}\right\}$ assuming that each $\sigma_{th}$ is drawn independently from a given distribution. In the simulations, we employed two probability distribution functions (pdf): (i) the Weibull pdf, whose two-parameter cumulative distribution function reads

(1) $$P_{\rho ,\lambda }\left(\sigma_{th}\right)=1-\exp\left[-{\left(\sigma_{th}/\lambda \right)}^{\rho }\right].$$

and (ii) a uniform distribution on the interval [0,1]. In Equation (1), the so-called Weibull index ρ refers to an amount of threshold disorder and λ scales a reference load. We assume that λ=1.

### 2.2. Load Transfer Rules Reflecting Substrate Rigidities

Multiple characteristics of array substrate as well as those corresponding to pillar fixations, conspire to regulate the load transfer processes. Specifically, since loads released by failing pillars are undertaken by the intact ones and pillar-to-pillar interactions pass through the substrate, then a correct rule of load transfer should be related to some mechanical properties of the substrate. Our rough model assumes that this rule is qualitatively linked to the substrate rigidity only. Such a simple assumption stems from the fact that immediately after loading, pillars start to interact elastically through stresses which are localized in the substrate. In order to choose a correct load transfer rule, we follow a power law relation $∼1/r^{\gamma }$ which expresses how the stress changes at the distance *r* from a damage location in a continuous and homogenous material. Our approach neglects thermal effects on the failure process [29,30].

A discrete analogue of the $∼1/r^{\gamma }$ stress-intensity drop, the so-called range variable (RV) load transfer rule [17,31,32,33], is suitable when dealing with cascading processes on networks. This is also the case of load-induced cascades of crushing pillars. In this scenario, we denote by $G_{0}$ the set of nodes occupied by intact pillars prior to loading. Once the load *F* is applied, the pillars start to break and form a finite sequence of *m* cascades of failures, where *m* depends on the load magnitude for a given array of pillars. Hence, we may split $G_{0}$ into two evolving sets, $G_{\mu }$ and $K_{\mu }$, which include nodes holding the intact and crushed pillars, respectively on *m* consecutive cascades, i.e.,

(2) $$\begin{array}{ccc} & & K_{\mu }\cap G_{\mu }=Ø\wedge K_{\mu }\cup G_{\mu }=G_{0},\mu =0,1,\cdots ,m, \end{array}$$

(3) $$\begin{array}{ccc} & & G_{0}\left(F=0\right)⟶G_{m}\left(F>0\right)\subset \cdots \subset G_{\mu }\left(F\right)\subset \cdots \subset G_{0}\left(F=0\right). \end{array}$$

If $G_{m}\left(F\right)\neq Ø$, then the array sustains the load *F*. According to the RV rule, a fraction $\Delta f_{j\leftarrow i}$ of load $f_{i}$ transferred from the failed pillar at node $i\in K_{\mu }$ to an accessible intact one located at $j\in G_{\mu }$ is defined as follows

(4) $$\Delta f_{j\leftarrow i}=\frac{Z_{i}}{|j-i{|}^{\gamma }}f_{i},$$

where ∣j−i∣ is the distance between crushed and intact pillars, and the normalisation factor $Z_{i}={\left(\sum_{j\in G_{\mu }}|j-i{|}^{-\gamma }\right)}^{-1}$ ensures that the applied load is conserved. The adjustable parameter γ≥0 should be tuned in accordance with the substrate rigidity. For this reason, we will explore how arrays of pillars behave under sudden loading in presence of RV(γ) rules with different values of γ, including the limits γ=0 and γ→∞ that correspond to perfectly rigid and maximally flexible substrates, respectively. It is worth mentioning that by varying the value of γ, the RV rule smoothly interpolates between the short range (γ→∞) and long range (γ=0) interactions among pillars. The short range limit corresponds to a so-called local load sharing (LLS) transfer rule, whereas the RV(γ=0) is identical to the rule known as global load sharing (GLS). From the mathematical point of view, the GLS rule is equivalent to a mean field approximation where any two pillars are assumed to be separated by the same distance. This, in turn, means that the GLS rule distributes the transferred load equally among intact pillars and thus it represents the least damaging load transfer protocol. On the other hand, the LLS rule allocates loads from failed pillars to their nearest intact neighbours only. Hence, it triggers the most harmful effects.

### 2.3. Survivability and Survival Function

Although there exists a formal definition of system survivability that is suitable in some areas of computer sciences, technology, or economy, there is no unique definition in a general sense [34]. In the context of our study, we define an array of pillars’ survivability as the capability of an array to sustain the applied load, although some fraction of pillars is down.

Along with this qualitative definition, we introduce a measure of survivability, namely the survival function *S*. For an array represented by ${\left\{\sigma_{th}\right\}}_{G_{0}}$, we define this function as the probability that $G_{0}$ evolves to a non-empty terminal set $G_{m}\left(f\right)$ when the load *f* is applied suddenly and the RV(γ) rule is in operation

(5) $$S\left(f,\gamma ,{\left\{\sigma_{th}\right\}}_{G_{0}}\right)=P\left(G_{0}⟶G_{m}\left(f,\gamma ,\left\{\sigma_{th}\right\}\right)\neq Ø\right)$$

A computational scheme that enables us to estimate the function (5) is specified below.

### 2.4. Computational Scheme

With the aim of correctly estimating the survival function S(f,γ), we have to collect large data sets corresponding to values of γ that grow from 0 to a certain $\gamma^{*}<\infty$, where $\gamma^{*}$ is settled indirectly via the condition that the results of simulations with the RV$\left(\gamma^{*}\right)$ rule are statistically indistinguishable from those obtained in the presence of the LLS rule. The case of the LLS rule is also taken into account. Furthermore, when $\left\{\sigma_{th}\right\}$ are governed by the Weibull distribution, we multiplicate simulations by varying the Weibull index ρ in order to pass from a strong to a weak disorder in pillar-strength thresholds.

Our simulations are arranged in the following way. For an array with a given number *N* of pillars whose the set of nodes is $G_{0}$ and a specified value of ρ, we store a simple sample Σ(ρ) comprising *M* ordered sets ${\left\{\sigma_{th}\left(\rho \right)\right\}}_{G_{0}}^{\left(k\right)},1\leq k\leq M$, each representing an array by *N* pillar-strength thresholds generated according to Equation (1). Additionally, we build a similar sample $\Sigma_{u}$ with ${\left\{\sigma_{th}\right\}}_{G_{0}}^{\left(k\right)}$ drawn from the uniform distribution on [0,1].

Then, having Σ, a value of γ, and a load *f*, we simulate the sudden loading with the RV(γ) rule in operation. Finally, we obtain all $G_{m}^{\left(k\right)}\left(f,\gamma ,{\left\{\sigma_{th}\right\}}_{G_{0}}^{\left(k\right)}\right)$ that refer to the chosen sample.

### 2.5. Survival Function Estimate

To estimate the survival function (5), we rely on the fact that our sample is simple and, hence, each individual ${\left\{\sigma_{th}\right\}}_{G_{0}}$ appears in the sample equally likely. This means that the probability of sustaining a given load becomes

$$P=\frac{\mathrm{number}\;\mathrm{of}\;\mathrm{arrays}\;\mathrm{in}\;\mathrm{the}\;\mathrm{sample}\;\mathrm{that}\;\mathrm{sustain}\;\mathrm{the}\;\mathrm{load}}{\mathrm{number}\;\mathrm{of}\;\mathrm{arrays}\;\mathrm{in}\;\mathrm{the}\;\mathrm{sample}\;}$$

and we may use the above approach to estimate the survival function *S*.

For a given N,γ,f and a chosen sample Σ, we simulate the sudden loading on all ${\left\{\sigma_{th}\right\}}_{G_{0}}\in \Sigma$ to obtain the corresponding terminal sets $G_{m}^{\left(k\right)}\left(f,\gamma ,\left\{\sigma_{th}\right\}\right),1\leq k\leq M$. Finally, the survival function estimator $\hat{S}$ is given by the fraction of arrays in Σ whose terminal sets are non-empty, i.e.,

(6) $$\hat{S}\left(f,\gamma ,{\left\{\sigma_{th}\right\}}_{G_{0}}\right)=\frac{1}{M}\sum_{k=1}^{M}H\left(|G_{m}^{\left(k\right)}\left(f,\gamma ,{\left\{\sigma_{th}\right\}}_{G_{0}}^{\left(k\right)}\right)|\right)$$

where ∣G∣ denotes the number of elements in G, and H(·) is the Heaviside step function, i.e., H(x)=1 if x>0; otherwise, it returns to zero. The estimator (6) will also be referred to as an empirical survival function.

## 3. Results and Discussion

We have carried out a substantial number of simulations of the model specified in Section 2 to collect data necessary to estimate the survival function correctly. We have gathered data for different values of N,ρ by varying load *f* in the presence of RV(γ) with γ spanned over the interval $\left[0,\gamma^{*}∼10\right]$. Separately, we have also gathered data referring to the large γ limit. Specifically, we have drawn $\left\{\sigma_{th}\left(\rho \right)\right\}$ from the Weibull distributions with $\rho \in \left\{2,3,\cdots ,10\right\}$ to simulate arrays with number of pillars $N\in \left\{40\times 40,\cdots ,250\times 250\right\}$. The same values of *N* were used for uniformly distributed σs. All simulations were conducted over samples comprising $M=10^{4}$ elements each.

Two assumptions are valid through all simulations: (i) the load transfer is an almost instantaneous process that happens simultaneously and (ii) pillars are loaded uniformly, i.e., initial load $f_{i\in G_{0}}$ on each pillar is equal to f=F/N.

### 3.1. Empirical Survival Functions Referring to the Range-of-Load-Transfer Limits

We have mentioned in Section 2.2 that the RV(γ=0) is equivalent to the GLS rule, which engages uniformly all intact pillars in capturing loads transferred from crushed pillars. Through this, the GLS as the least damaging rule enables us to estimate the upper bound of strength of an array represented by ${\left\{\sigma_{th}\right\}}_{G_{0}}$. On the other hand, in the limit γ→∞, the RV(γ) converges to the LLS rule, which, by activating only the nearest neighbours of crushed pillars, provokes the most adverse effects on the system. When the LLS rule operates it yields the lower bound of overall strength of the same array.

Although the GLS and the LLS rules are rather unrealistic, we analyse data resulting from simulations with them in order to have access to a corresponding system’s strength limits.

#### 3.1.1. Survival Function under the GLS Rule, RV(γ=0)

**Figure 1:**
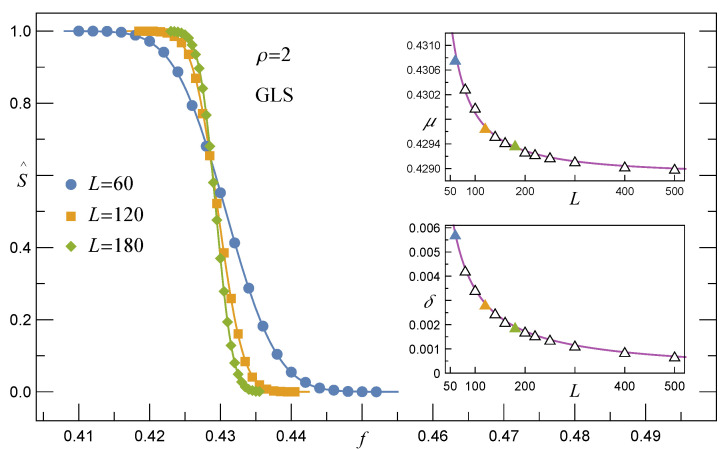
The empirical survival function ${\hat{S}}_{\mathrm{GLS}}$ for arrays with L×L pillars and $\left\{\sigma_{th}\right\}$ governed by the Weibull distribution. Continuous lines follow Equation (7) with parameters μ and δ defined, respectively, by Equations (8) and (9).

Let us first analyse how the GLS rule affects the survivability of an array of pillars. In Figure 1, the empirical survival function is plotted for a growing number of pillars. Applying the Cramer–von Mises and Anderson–Darling goodness-of-fit tests, we were able to fit all data presented in Figure 1 by one family of functions, namely the complementary cumulative normal distribution, that takes the form

(7) $$\begin{array}{ccc} S_{\mathrm{GLS}}\left(f\right) & = & 1-\mathrm{CN}(f),\\ \mathrm{CN}(f) & = & \frac{1}{2}\mathrm{erfc}\left(-\frac{f-\mu }{\sqrt{2}\delta }\right). \end{array}$$

The above functional form of *S* depends on two parameters: the location μ and the scale δ. Both of these parameters turn out to be very accurately fitted by the mean critical load ${\bar{f}}_{c}$ and the standard deviation of distribution of $f_{c}$ computed for fibre bundles loaded quasi-statically with the GLS rule [35,36,37]. When the Weibull distribution generates ${\left\{\sigma_{th}\right\}}_{G_{0}}$, these parameters read

(8) $$\mu \left(\rho ,N\right)={\bar{f}}_{c}\left(\rho ,N\right)={\left(\rho e\right)}^{-\frac{1}{\rho }}\left[1+0.996N^{-2/3}{\left(\frac{e^{2/\rho }}{\rho }\right)}^{1/3}\right]$$

and

(9) $$\delta \left(\rho ,N\right)=\mathrm{sd}\left(N\right)=\left(\frac{1}{\sqrt{N}}\right)\rho^{-1/\rho }\sqrt{e^{-1/\rho }\left(1-e^{-1/\rho }\right)}.$$

For uniformly distributed ${\left\{\sigma_{th}\right\}}_{G_{0}}$, the corresponding location μ is given by [38]

(10) $$\mu \left(N\right)={\bar{f}}_{c}\left(N\right)=\frac{1}{4}\left(1+1.224N^{-2/3}\right).$$

All continuous lines in Figure 1 intersect in a close vicinity of one point. This common crossing point corresponds to the limit ${\bar{f}}_{c}^{\infty }=\lim_{N\to \infty }{\bar{f}}_{c}$. For arrays with Weibull distributed pillar-strength thresholds, this limit equals ${\left(\rho e\right)}^{-\frac{1}{\rho }}$, whereas for the uniform distribution on [0,1] we have ${\bar{f}}_{c}^{\infty }=1/4$; see Equations (8) and (10), respectively. Finally, in the limit of a very large number of pillars, $S_{\mathrm{GLS}}$ converges to the step function expressed with the help of the Heaviside function

(11) $$S_{\mathrm{GLS}}\left(f,\infty \right)=1-H\left({\bar{f}}_{c}^{\infty }\right).$$

#### 3.1.2. Survival Function under the LLS Rule, RV(γ→∞)

**Figure 2:**
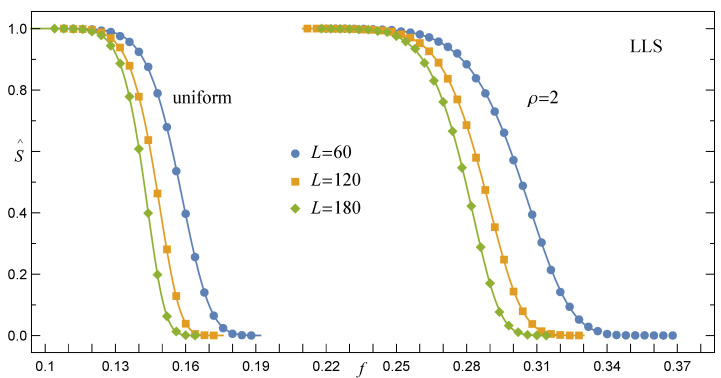
The empirical survival function ${\hat{S}}_{\mathrm{LLS}}$ of loaded arrays involving L×L pillars with respect to two distributions of $\left\{\sigma_{th}\right\}$. The solid lines represent Equation (12).

Empirical survival function ${\hat{S}}_{LLS}$ defined by Equation (6) and computed for arrays with a growing number of pillars is presented in Figure 2. Similarly to the already analysed ${\hat{S}}_{GLS}$, data points are also satisfactorily fitted in this case by one family of functions specified by the cumulative skew-normal distribution (CSN) [39]

(12) $$\begin{array}{ccc} S_{\mathrm{LLS}}\left(f\right) & = & 1-\mathrm{CSN}(f),\\ \mathrm{CSN}(f) & = & \frac{1}{2}\mathrm{erfc}\left(-\frac{f-\zeta }{\sqrt{2}\omega }\right)-2T\left(\frac{f-\zeta }{\omega },\theta \right), \end{array}$$

where the location (ζ), scale (ω), and shape (θ) are the parameters depending on *N* and $\left\{\sigma_{th}\right\}$. T(∘,∘) stands for Owen’s *T* function.

**Figure 3:**
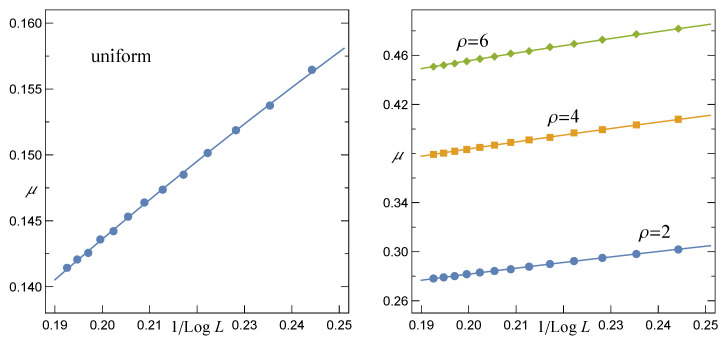
The expectation μ for arrays with L×L pillars and the LLS rule. The solid lines are drawn in accordance with Equation (14).

In accordance with the results of simulations, when *N* grows, the expectation value

(13) $$\mu =\zeta +\sqrt{\frac{2}{\pi }}\cdot \frac{\omega \theta }{\sqrt{\left(1+\theta^{2}\right)}}$$

that is related to the CSN(f) and displayed in Figure 3 approaches the following power-law relation

(14) $$\mu \left(N,\left\{\sigma_{th}\right\}\right)=\alpha \left(\left\{\sigma_{th}\right\}\right){\left(\frac{1}{\ln N}\right)}^{\beta (\left\{\sigma_{th}\right\})}$$

**Figure 4:**
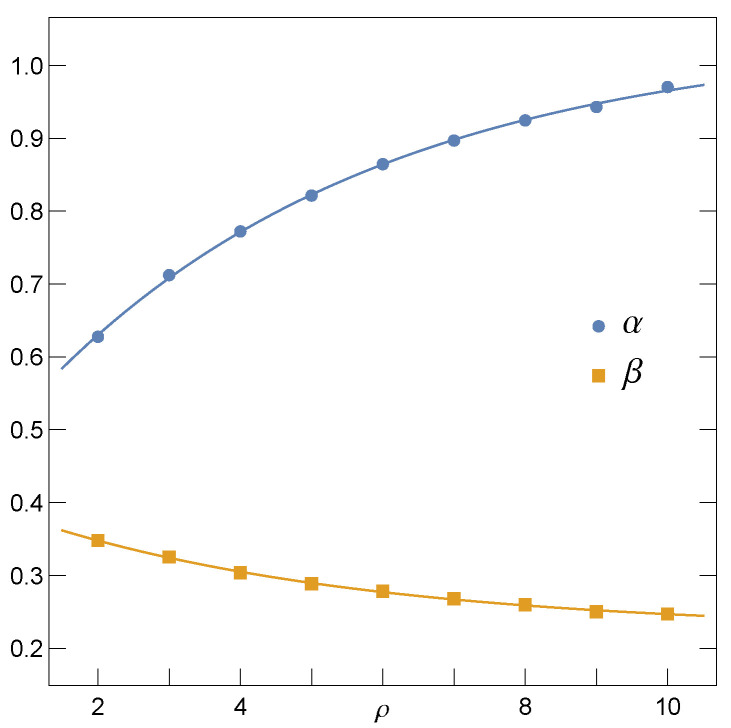
The parameters α and β of Equation (14) and their best fits defined correspondingly by Equations (15) and (16).

The same relation, however, represents the best fit to the mean ultimate strength ${\bar{f}}_{c}$ of quasi-statically loaded multicomponent systems represented by the Fibre Bundle Model with the LLS rule [26,38,40,41,42]. Specifically, when $\left\{\sigma_{th}\right\}$ are distributed uniformly over [0,1], the corresponding coefficients are: α≃0.380,β≃0.423. We have also noticed that if $\left\{\sigma_{th}\right\}$ are assigned to pillars according to the Weibull distribution (1), then α(ρ) and β(ρ) are best fitted by the exponential functions

(15) $$\begin{array}{c} \alpha \left(\rho \right)=A_{\alpha }-B_{\alpha }\cdot \exp\left(-\lambda \cdot \rho \right), \end{array}$$

(16) $$\begin{array}{c} \beta \left(\rho \right)=A_{\beta }+B_{\beta }\cdot \exp\left(-\lambda \cdot \rho \right), \end{array}$$

where: $A_{\alpha }=1.043\pm 0.024,B_{\alpha }=0.627\pm 0.017,A_{\beta }=0.224\pm 0.009,B_{\beta }=0.189\pm 0.008$ and λ=0.210±0.033. In Figure 4, we display the above best fits together with corresponding data points.

### 3.2. Empirical Survival Function for Intermediate Ranges of Load Transfer

We have gathered large data sets concerning the empirical survival function (6) of arrays with a growing number of pillars in the presence of RV(γ) rule with increasing values of γ. A careful analysis of the data indicates that when loads are transferred according to RV(0<γ<∞), the survival function is satisfactorily modelled by the function introduced in Equation (12), namely

(17) $$\begin{array}{ccc} S_{\mathrm{RV}}\left(f,\gamma \right) & = & 1-\mathrm{CSN}(f,\gamma ),\\ \mathrm{CSN}(f,\gamma ) & = & \frac{1}{2}\mathrm{erfc}\left[-\frac{f-\zeta (\gamma )}{\sqrt{2}\omega \left(\gamma \right)}\right]-2T\left[\frac{f-\zeta (\gamma )}{\omega (\gamma )},\theta \left(\gamma \right)\right]. \end{array}$$

We have explicitly written that the parameters ζ,ω and θ depend on the range of load transfer through γ, while their obvious dependence on $\left\{\sigma_{th}\right\}$, is omitted for the sake of clarity.

**Figure 5:**
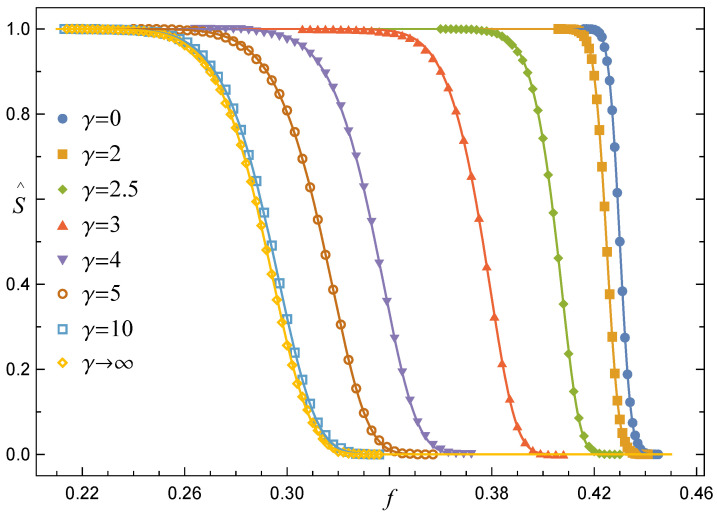
The evolution of the survival functions (curves) for different γ and N=100×100. Pillar-strength thresholds are generated according to the Weibull distribution with ρ=2.

In Figure 5, we present a collection of empirical survival functions ${\hat{S}}_{\mathrm{RV}}\left(f,\gamma \right)$ together with the corresponding best fits, Equation (17), ordered from left to right by decreasing values of γ. Although these functions correspond to a sample of arrays with a moderate number of pillars, two characteristic features are clearly seen: (i) S(f,γ≲2) behaves similarly to ${\hat{S}}_{\mathrm{GLS}}\left(f\right)$, whereas for γ≳3 the shape of $\hat{S}$ resembles that one of ${\hat{S}}_{\mathrm{LLS}}\left(f\right)$ and (ii) with growing γ locations of $\hat{S}\left(f\right)$ are shifted towards decreasing values of *f*.

**Figure 6:**
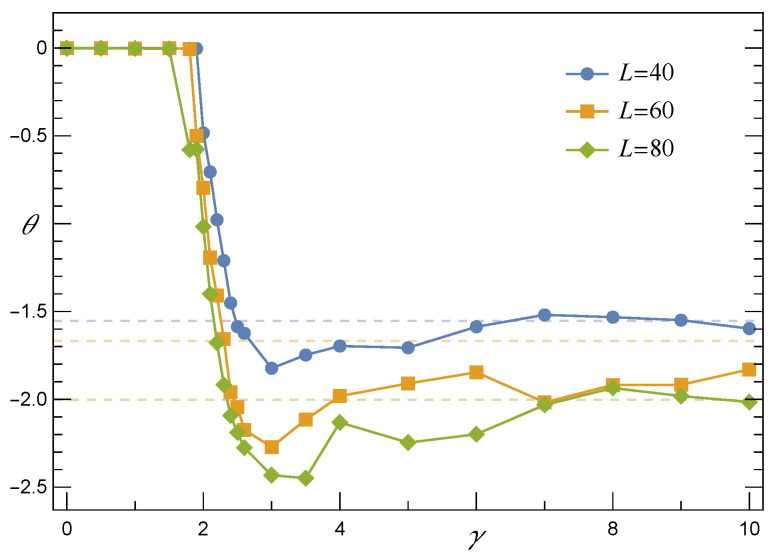
The shape parameter θ as function of γ for arrays of N=L×L pillars with $\left\{\sigma_{th}\right\}$ distributed uniformly over [0,1]. The dashed lines correspond to the respective large γ limits.

**Figure 7:**
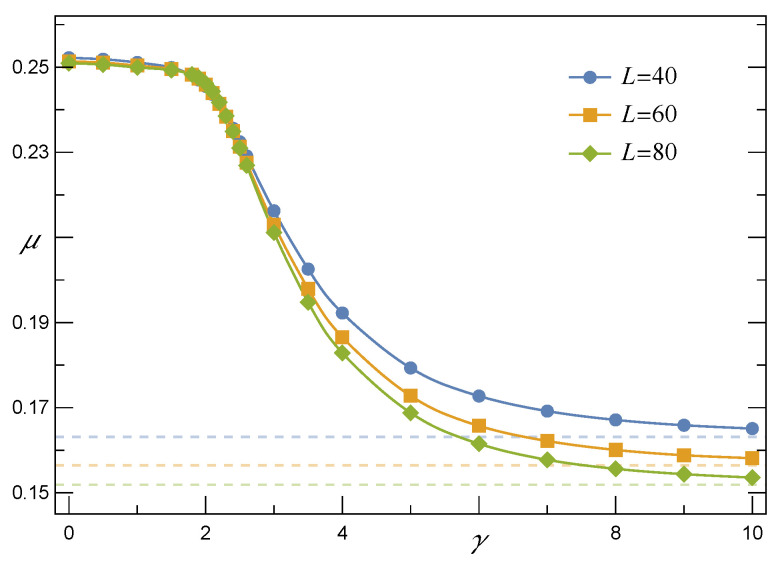
The expectation μ computed according to Equation (13) in reference to the survival function $S_{\mathrm{RV}}\left(f,\gamma \right)$ defined by Equation (17). Other parameters are the same as in Figure 6.

A closer look at Figure 6 and Figure 7 sheds light on the above-mentioned features. First, consider the shape parameter θ which is displayed in Figure 6. When γ stays in the range $\left[0,\hat{\gamma }\right]$ with $\hat{\gamma }∼2$, then $\theta (\gamma ≲\hat{\gamma })\approx 0$. It is seen from Equation (17) that the parameter θ only appears as the argument of the Owen’s T(∘,θ) function. By definition, however, T(∘,θ) becomes infinitesimally small when θ vanishes. Consequently, $S_{\mathrm{RV}}\left(f,\gamma \right)$ turn out to be effectively expressed by the cumulative skew-normal distribution with zero skewness

$$\mathrm{CSN}\left(f,0\leq \gamma \leq \hat{\gamma }\right)\approx \frac{1}{2}\mathrm{erfc}\left[-\frac{f-\zeta }{\sqrt{2}\omega }\right]=\mathrm{CN}\left(f\right).$$

Since the skewness vanishes in this range of γ, then the skew-normal distribution effectively becomes the standard normal one and the resulting survival function resembles $S_{\mathrm{GLS}}$. Moreover, the presence of a mean-field-like effective pillar-to-pillar interaction is supported by empirical values of μ that were computed according to Equation (13) and are presented in Figure 7. These values refer to CSN(f,γ) of Equation (17). It is seen in Figure 7 that if γ≲2.2, then the values of μ(γ) related to arrays with different numbers of pillars converge with each other. This vanishing system-size-dependence justifies the applicability of mean-field approximation and indicates that loads are transferred among pillars according to the GLS-like rule. On the other hand, when γ>3, the system-size-effect becomes visible. Hence, in the large *N* limit the mean μ(N,γ) approaches the power-law expression given by Equation (14).

**Figure 8:**
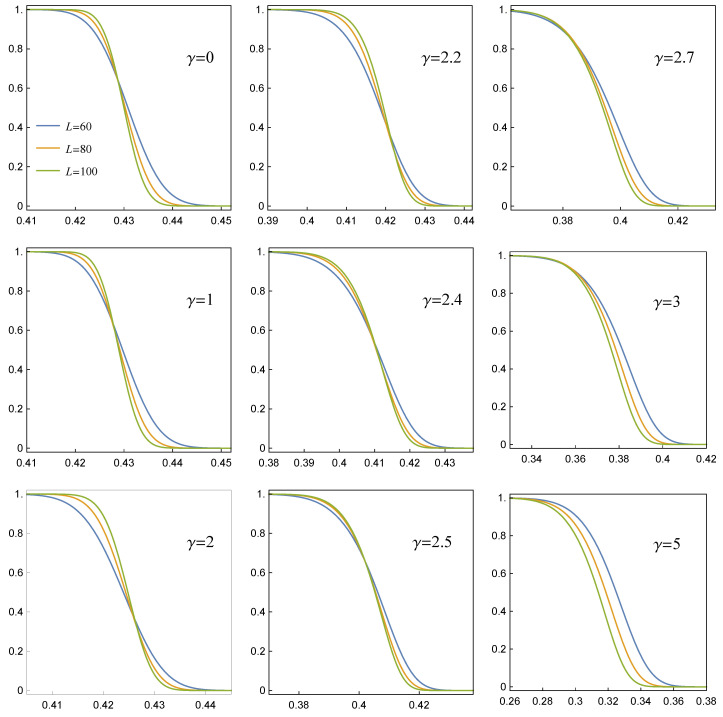
Fitted survival function $S_{\mathrm{RV}}\left(f,\gamma \right)$, see Equation (17), for arrays with L×L pillars and $\left\{\sigma_{th}\right\}$ drawn from the Weibull distribution with ρ=2.

From the above discussion, we may conclude that for small value of γ≲2.2 the survival function behaves like $S_{\mathrm{GLS}}$. Then, $S_{\mathrm{RV}}$ gradually converts to a $S_{\mathrm{LLS}}$-like survival function when a growing γ passes through a short segment [2.2,3]. In order to see this conversion of $S_{\mathrm{RV}}$ in detail, we have assembled data corresponding to samples with different values of *N* and have drawn the resulting survival functions in Figure 8.

Only the best fits given by Equation (17) are shown in Figure 8 as a matter of transparency. When γ≤2.4, the lines that represent arrays with different *N* intersect in a close vicinity of one point. This resembles the intersection point seen in Figure 1 that refers to the GLS rule. In contrast, lines stop crossing if γ≥2.7 and their positions together with mutual order reflect those corresponding to $S_{\mathrm{LLS}}$ presented in Figure 2.

It is worth noting that the segment [2.4,2.7] is shifted towards higher values of γ with respect to its counterpart [2,2.2] [17], which is related to quasi-statically loaded systems [31,32,33]. This can be explained as follows. It is seen in Figure 5 that when we take a certain *f* and two surviving functions $S\left(f,\gamma_{1}\right)\neq S\left(f,\gamma_{2}\right)$, then $S\left(f,\gamma_{1}\right)>S\left(f,\gamma_{2}\right)\Rightarrow \gamma_{1}<\gamma_{2}$. On the other hand, when a given ensemble of arrays is loaded quasi-statically up to a certain load $\tilde{f}$, then the fraction ${\hat{S}}_{q-\mathrm{static}}\left(\tilde{f}\right)$ of surviving arrays would be greater than ${\hat{S}}_{\mathrm{sudden}}\left(\tilde{f}\right)$ which results from the load $\tilde{f}$ being applied suddenly. Hence, having the same rule RV(γ) in both loadings, we obtain the above-mentioned shift.

### 3.3. Fraction of Surviving Pillars

Our main concern in this paper is the survival function *S*. By definition, however, *S* also holds hidden information about surviving pillars. In order to extract this information, we consider a sample of arrays with pillars specified by ${\left\{\sigma_{th}\right\}}_{G_{0}}$. When the sample is engaged in supporting the load *f*, we should know not only what the fraction of arrays is that will survive but also how large the fraction (*U*) of intact pillars will be accessible for further applications. To be precise, even though the empirical survival function (6) provides information about a fraction of arrays which are able to sustain suddenly applied load *f*, the function $\hat{S}\left(f\right)$ itself gives us no information about the quantities of intact pillars that work to bear the load *f*.

With this in mind, we define and compute the mean fraction $\bar{U}$ of surviving pillars by adopting the same arguments as those presented in Section 2.5, i.e.,

(18) $$\bar{U}\left(f,\gamma ,{\left\{\sigma_{th}\right\}}_{G_{0}}\right)=\frac{1}{M\cdot N}\sum_{k=1}^{M}|G_{m}^{\left(k\right)}\left(f,\gamma ,{\left\{\sigma_{th}\right\}}_{G_{0}}^{\left(k\right)}\right)|.$$

**Figure 9:**
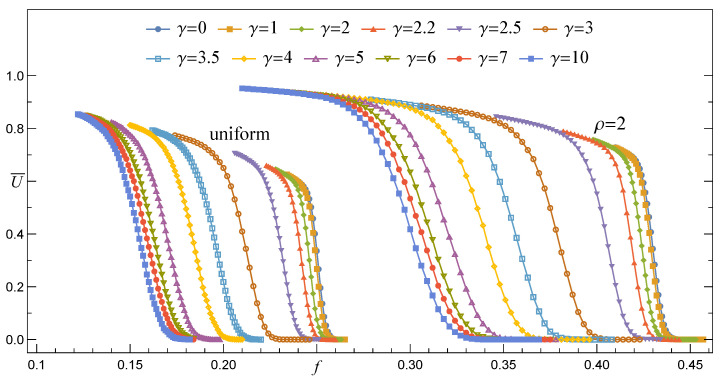
The variations of mean fraction of surviving pillars $\bar{U}\left(f,\gamma \right)$ are displayed for 0≤γ≤10 when $\left\{\sigma_{th}\right\}$ are distributed uniformly over [0,1] (left bunch of data points) or according to the Weibull pdf with ρ=2 (right bunch of data points). The continues lines follow Equation (20) with parameters estimated from simulations.

Below, we present data that relate the mean fraction of surviving pillars to model parameters. As an example, in Figure 9, we plot $\bar{U}\left(f,\gamma \right)$ for an array with 80×80 pillars including two distributions of pillar-strength thresholds and assuming that 0≤γ≤10.

An obvious feature seen in Figure 9 concerns the upper envelope of $\bar{U}$; let us denote it by ψ. Independently of distributions of $\left\{\sigma_{th}\right\}$, the envelope coincides with $\bar{U}\left(f,\gamma =\;0\right)$ and thus can be determined from data corresponding to RV(γ=0), i.e., to the GLS rule of load transfer; see Section 3.1. Although the data displayed in Figure 9 refer to a particular value of *N*, the same feature holds for any number of pillars.

Since $\bar{U}$ and $\hat{S}$ are closely related, it is worth identifying a direct link between them. For this purpose we have to establish the best fit $\psi_{N}\left(f,\left\{\sigma_{th}\right\}\right)$ to the above-mentioned envelope of $\bar{U}\left(f,\gamma ,\left\{\sigma_{th}\right\},N\right)$. By applying the maximum likelihood procedure, we have found that $∼f^{b}$ is the best fit to $\psi_{N}\left(f,\left\{\sigma_{th}\right\}\right)$, i.e.,

(19) $$\psi_{N}\left(f,\left\{\sigma_{th}\right\}\right)=1-a_{N}\left(\left\{\sigma_{th}\right\}\right)\cdot f^{b_{N}\left(\left\{\sigma_{th}\right\}\right)}$$

where $a_{N}\left(\left\{\sigma_{th}\right\}\right),b_{N}\left(\left\{\sigma_{th}\right\}\right)$ are coefficients that should be computed from data related to *N* and to pillar-strength thresholds.

A rigorous comparison of data collected from simulations with data provided by Equations (17) and (19) allows us to express the mean fraction of surviving pillars in the following way

(20) $${\bar{U}}_{N}\left(f,\gamma ,\left\{\sigma_{th}\right\}\right)=\psi_{N}\left(\left(f,\left\{\sigma_{th}\right\}\right)\right)\cdot {\hat{S}}_{N}\left(f,\gamma ,\left\{\sigma_{th}\right\}\right)$$

**Figure 10:**
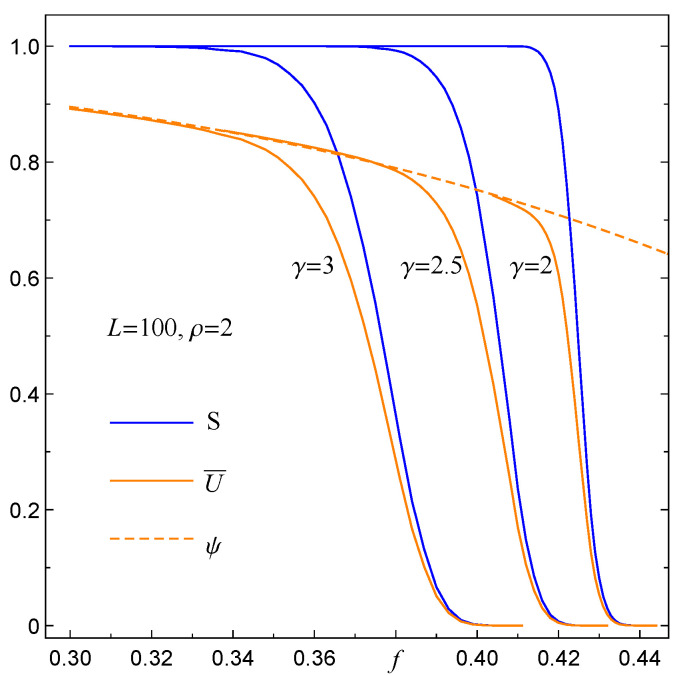
The link between $\bar{U}\left(f,\gamma \right)$, S(f,γ) and the envelope function ψ(f) expressed by Equation (21).

The above relation shows that the mean fraction of surviving pillars and the surviving functions are linked via the envelope function (19). Specifically, for the Weibull pdf, we have the following relation:

(21) $${\bar{U}}_{N}\left(f,\gamma ,\rho \right)=\left[1-a_{N}\left(\rho \right)\cdot f^{b_{N}\left(\rho \right)}\right]\cdot {\hat{S}}_{N}\left(f,\gamma ,\rho \right),$$

which is displayed in Figure 10 for an exemplary sample of arrays, each with 100×100 pillars and $\left\{\sigma_{th}\right\}$ drawn with the Weibull index ρ=2.

More detailed information in reference to locations of intact pillars within consecutive cascades of failures is out of the scope of this paper. We will address this issue in future works.

## 4. Summary

We have employed the range variable fibre bundle model to study the survivability of arrays with vertical pillars located at nodes of square substrates and subjected to axial loads applied suddenly. In our approach, amplitudes of loads transferred from the crushed to intact pillars decay with distance *r* as $r^{-\gamma }$, where γ qualitatively reflects the effective elasticity of the substrate. By this, growing values of γ span a variety of substrates from perfectly rigid to flexible ones.

Based on simulations, we were able to discriminate between rules of load transfer RV(γ) with γ≤2.4 and those with γ≥2.7. When the former rules are present, a loaded array behaves as the bundle of pillars located on a rigid substrate with a long-range GLS-like rule of load transfer. This is in contrast to the latter rules which effectively act as an effective short-range LLS-like load transfer rule. Our simulations and collected data do not allow us to classify rules which correspond to 2.4<γ<2.7.

We have also found that the empirical survival functions $\hat{S}\left(f\right)$, defined as the probability that specified arrays with pillars sustain a given load *f* despite of partial damages, are very accurately fitted by one family of distributions, namely the family of complementary cumulative skew-normal distributions; see Equation (17).

We have mentioned in the Introduction section that micropillar arrays are in the core of different kinds of nanodevices such as, e.g., piezoelectric nanogenerators. It remains to be investigated whether and how our approach can be tailored to analyse pillar failures in such devices.

We are conscious of the fact that the results of our work rely on statistically founded modelling that requires extensive numerical simulations. This is, however, justified by the complexity of the problem that prevents an analytically tractable approach that goes beyond mean-field approximations, such as the GLS fibre bundle model. 

## Data Availability

Not applicable.
